# Human Liver Cells Expressing Albumin and Mesenchymal Characteristics Give Rise to Insulin-Producing Cells

**DOI:** 10.1155/2011/252387

**Published:** 2011-08-24

**Authors:** Irit Meivar-Levy, Tamar Sapir, Dana Berneman, Tal Weissbach, Sylvie Polak-Charcon, Philippe Ravassard, Andreas G. Tzakis, Eytan Mor, Camillo Ricordi, Sarah Ferber

**Affiliations:** ^1^Sheba Regenerative Medicine, Stem cells and Tissue engineering Center, Sheba Medical Center, Tel-Hashomer 52621, Israel; ^2^Diabetes Research Institute, University of Miami Leonard M. Miller School of Medicine, 1450 NW 10th Avenue, Miami, FL 33136, USA; ^3^Department of Human Genetics and Molecular Medicine, Sackler School of Medicine, Tel-Aviv University, 69978 Tel-Aviv, Israel; ^4^Obstetrics and Gynecology Department, Sapir Medical Ctrter 44281 Kfar Saba, Israel; ^5^The Institute for Pathology, Sheba Medical Center, 52621 Tel-Hashomer, Israel; ^6^Biotechnology and Biotherapy Group, Centre de Recherche, Institut du Cerveau et de la Moelle CNRS UMR7225, INSERM UMRS795, Université Pierre et Marie Curie, 75005 Paris, France; ^7^The Miami Transplant Institute, University of Miami Leonard M. Miller School of Medicine, 1450 NW 10th Avenue, Miami, FL 33136, USA; ^8^Rabin Medical Center, Beilinson Campus, 49100 Petah-Tiqva, Israel

## Abstract

Activation of the pancreatic lineage in the liver has been suggested as a potential autologous cell replacement therapy for diabetic patients. Transcription factors-induced liver-to-pancreas reprogramming has been demonstrated in numerous species both *in vivo* and *in vitro*. However, human-derived liver cells capable of acquiring the alternate pancreatic repertoire have never been characterized. It is yet unknown whether hepatic-like stem cells or rather adult liver cells give rise to insulin-producing cells. Using an *in vitro* experimental system, we demonstrate that proliferating adherent human liver cells acquire mesenchymal-like characteristics and a considerable level of cellular plasticity. However, using a lineage-tracing approach, we demonstrate that insulin-producing cells are primarily generated in cells enriched for adult hepatic markers that coexpress both albumin and mesenchymal markers. Taken together, our data suggest that adult human hepatic tissue retains a substantial level of developmental plasticity, which could be exploited in regenerative medicine approaches.

## 1. Introduction

A cure for type 1 diabetes mellitus depends on replenishing functional insulin-producing cells. However, the limited supply of pancreatic islets from cadaver donors and the need for life-long immune suppression makes pancreas or pancreatic islet allotransplantation impractical for the vast majority of patients. This hurdle has led to a search for new alternate sources of insulin-producing cells or tissues [1–3]. A challenging approach to generating surrogate *β*-cells for cell replacement therapy in diabetes is the direct reprogramming of liver cells into insulin-producing cells. Liver and pancreatic cells share a common developmental origin, making liver cells good candidates for manipulation into *β*-like cells [4]. Both tissues may share a common population of progenitor cells [5]. For example, cells with properties virtually identical to those of hepatic oval cells can also emerge in the pancreas, especially after the ablation of acinar cells [6]. Upon transplantation, these pancreas-derived oval cells can differentiate into functional hepatocytes and bile ducts [7]. The reciprocal conversion of rodent [8–13] and human [14–18] liver cells into pancreatic endocrine cells by transdifferentiation or direct cellular reprogramming has also been described. Liver cells have been induced to differentiate into insulin-producing cells by ectopic expression of pancreatic transcription factors, the best studied of which is PDX-1, a key regulator of pancreatic development and insulin expression in adult pancreatic beta cells [8, 10, 14, 16–19]. However, whether transcription factor-induced reprogramming primarily occurs in “stem-like” pluripotent cells or rather adult cells can directly give rise to committed cells of alternate lineages is questionable and presently analyzed. 

Adult liver contains several populations of cells, including hepatocytes, cholangiocytes, endothelial stellate cells, and bone marrow- (BM-) derived cells [20–22]. Activation of the pancreatic lineage in mice *in vivo* has been reported to occur in several areas of the intact organ [8, 11, 23, 24]. PDX-1-induced insulin production in mouse livers *in vivo* appears to occur mainly in the parenchyma of the liver around the central veins [8, 11, 25]. On the other hand, NEUROD1 and betacellulin-induced insulin production occurs mainly in cells close to the hepatic capsule [24]. NGN-3 and betacellulin induce the transdetermination of parenchymal hepatocytes and hepatic progenitor cells and possibly endoderm-derived oval cells in periportal areas of the liver [26]. 

The aim of the present study was to characterize the human-derived liver cells capable of giving rise to insulin-producing cells and determine whether they originate from mature or hepatic progenitor cells. The definite characteristics of hepatic progenitor cells which populate the adult human organ is controversial; however, there is a wide agreement that all these populations express the epithelial marker, EpCAM [27–29]. We generated *in vitro* primary cultures of liver cells derived from different human donors that already demonstrated a capacity to reprogram along the endocrine pancreatic and *β*-cell-like lineages by ectopic expression of pancreatic transcription factors [14–16, 30]. The origin of the induced insulin-positive cells in proliferating cultures of adult human liver cells is not clear, because the original liver cell morphology is altered and the cells undergo massive dedifferentiation, which is further augmented by ectopic PDX-1 expression and the reprogramming process itself [30]. Here, we characterize the cells in the adherent, proliferating cultures derived from adult human liver and demonstrate their mesenchymal-like characteristics. Using a genetic cell lineage tracing for albumin, we demonstrate that the cells coexpress both mesenchymal and adult hepatic markers, but none of the cells express EpCAM. The mesenchymal-like cells that originate from cells expressing albumin give rise to insulin expression upon ectopic PDX-1 expression. 

## 2. Material and Methods

### 2.1. Human Liver Cells

Adult human liver tissues were obtained from 3 different liver transplantation surgeries from 4–10-years-old children and 8 individuals over forty years old. Liver tissues were used with approval from the Committee on Clinical Investigations (institutional review board).

Isolation of human liver cells was performed as previously described [16, 31]. Briefly, the cells were digested by 0.03% Collagenase type I (*Worthington Biochemical Corp.*, NJ) and cultured in Dulbecco's minimal essential medium (1 gr/L glucose) supplemented with 10% FCS, 100 U/mL penicillin, 100 *μ*g/mL streptomycin and 250 ng/mL amphotericin B (*Biological Industries*, Israel). The medium was changed daily during the first three days in order to remove nonadherent cells. Ninety percent confluent cultures were split using trypsin-EDTA. The cells were kept at 37°C in a humidified atmosphere of 5% CO_2_ and 95% air.

### 2.2. Lentivirus Vector Construction and Virus Production

The pTrip albumin promoter (410) nlsCRE DeltaU3 (ALB-Cre) vector was generated by removing with *Bam*HI and *Xho*I of the enhanced green fluorescent protein (eGFP) coding region from the pTrip ALB eGFP DeltaU3 vector, which contains a fragment of the rat albumin promoter from −423 to −23 relative to the transcription start site. The resulting linearized plasmid was blunt-ended with DNA polymerase I Klenow fragment. The reading frame A Gateway cassette (Gateway Conversion kit; Invitrogen) was next ligated to the blunt-ended vector according to the manufacturer's instructions, generating a pTrip ALB rfa-Gateway DeltaU3 destination vector. The nlsCRE fragment was amplified by PCR from a plasmid [32] provided by Guilan Vodjdani (Hospital de la Pitie′, Salpetriere, Paris) using the forward primer 5′ CACCAGATCTATGCCCAAGAAGA.

AGAGG-3′ and the reverse primer 5′-CTCGAGCTAATCGCCATCTTC-3′, and the resulting PCR product was cloned into the pENTR/D/TOPO plasmid (Invitrogen) to generate an nls-CRE entry clone. Both destination vector and entry clone were used for *in vitro* recombination using the LR clonase II system (Invitrogen) according to the manufacturer's instructions. The reporter vector was constructed as previously reported [33].

Virus particles were produced in 293T cells after pCMVdR8.91 and pMD2.G vectors cotransfection. The culture medium was harvested 36–48 h later.

### 2.3. Viral Infection

Lentiviruses infection was performed 24 hours after plating; liver cells were washed with PBS and infected with a 1 : 1 mixture of the two viruses at multiplicity of infection (MOI) 3 : 1 in growth media containing 8 ng/mL polybrene overnight. The medium was then replaced with culture medium, and the cells were refed twice a week and split 1 : 3 once a week. The percentiles of eGFP and DsRed2 positive cells were analyzed using a Beckman Coulter FC500 flow cytometer or FACS Calibur, using the CellQuest program. 

Adenoviral infection of *Ad-CMV-PDX-1* (1000 MOI) was preformed as previously reported [14, 16, 30].

### 2.4. Animal Studies

All animals were maintained and animal experiments were carried out under the supervision and guidelines of the Sheba Medical center Institutional Animal Welfare Committee (177/2002).

Cells at passage 4, were harvested, washed twice with sterile PBS, counted, and resuspended in Matrigel (BD Biosciences). Six-week-old female athymic nude mice were injected subcutaneously in both flanks with human liver cells at density of 1 × 10^6^ viable cells/100 *μ*L as previously described [34]. Five mice were used in each group. Tumor size was measured with a linear caliper for up to 17 weeks.

### 2.5. Flow Cytometry

Liver-derived cells were harvested and washed with flow cytometry buffer consisting of 1% BSA and 0.1% sodium azide (*Sigma*, St. Louis, Mo,USA) in phosphate buffered saline (*Invitoge*n, Carlsbad, Calif,USA). For the cell surface antigen detection, approximately 10^5^ cells labeled with conjugated monoclonal antibodies. Intracellular staining was preformed using Intracellular Staining Flow Assay Kit (*Imgenex*, San Diego, Calif, USA) following manufacturer's instruction. Control samples included unstained cells, isotype antibody stained cells, and single fluorochrome-stained cells. The antibodies used in this study are listed in supplemental material data 1.

 The cells were analyzed using a Beckman Coulter FC500 flow cytometer or FACS Calibur, using the CellQuest program.

### 2.6. Cell Sorting

Three weeks after lentiviruses infection the labeled liver-derived cells were sorted using a fluorescence-activated cell sorter (FACS) (Aria cell sorter; Becton Dickinson, San Jose, Calif, USA) with a fluorescein isothiocyanate filter (530/30 nm) for eGFP and a Pe-Texas Red filter (610/20 nm) for DsRed2.

### 2.7. *In Vitro* Adipogenic and Osteogenic Differentiation

Following manufacturer's instructions (Human Mesenchymal Stem Cell Functional Identification Kit, *R&D Systems*, Minneapolis, Minn,USA), human liver-derived cells at passage 4 were plated on cover slips at 2,000 cells/cm^2^ in 6-well tissue culture-treated plates in the presence of the adipogenic or osteogenic supplements provided by the company. The appropriate supplemented medium was changed twice per week. After 14 days in culture, the adipogenic culture formed adipogenic-like vacuoles. The plates were fixed with 4% paraformaldehyde for 20 minutes and stained with Oil Red O (*Sigma*). The osteogenic differentiation cultures were incubated for 21 days and fixed and stained with 1% Alizarin Red solution pH 4.1 (Sigma). The calcium deposits were stained orange-red. Slides were imaged under a Leica DMLB microscope using the Leica Application Suite version 2.7.1 R1 software.

### 2.8. RNA Isolation, RT and RT-PCR Reactions

Total RNA was isolated, cDNA was prepared and amplified as described previously [8, 16]. Quantitative real-time RT-PCR was performed using ABI StepOnePlus (*Applied Biosystems*, Calif, USA) as described previously [14, 16, 30]. The primer pairs and annealing temperatures listed in supplemental material data 2. 

### 2.9. Immunofluorescence

Human liver cells treated were plated on glass cover slides in six-well culture plates. Forty-eight hours later, the cells were fixed and stained as described previously [16]. The antibodies used in this study are listed in supplemental material data 1.

The slides were analyzed using a fluorescent microscope (Provis, Olympus).

### 2.10. Statistical Analyses

Statistical analyses were performed using two-sample Students *t*-test assuming unequal variances.

## 3. Results

### 3.1. Characterization of Human Liver-Derived Cells Cultured *In Vitro*

**Figure 1:**
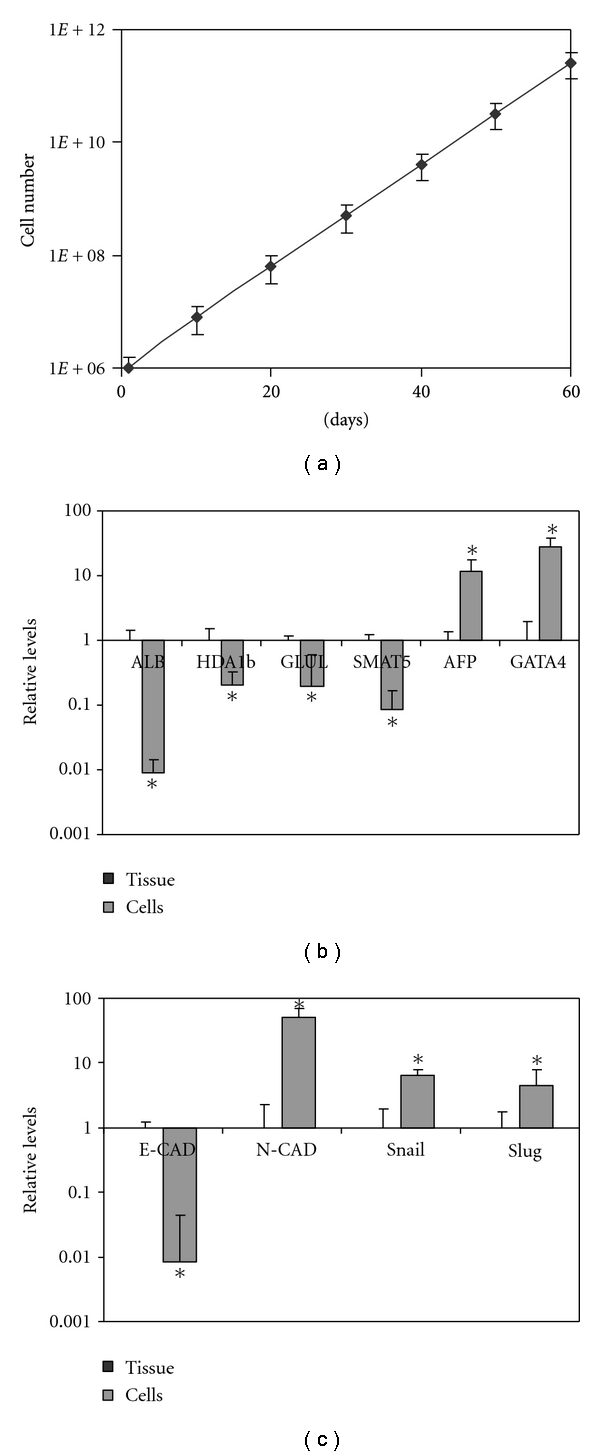
Proliferating adult human liver cells in culture undergo hepatic dedifferentiation associated with EMT marker expression. (a) Growth curve of adult human liver cells supplemented with exendin-4 (5 nM) [11]. (b) Quantitative RT-PCR analysis of hepatic (albumin, ADH1b, GLUL, SMAT5, and AFP), endodermal (GATA4) and (c) EMT (E-CAD, N-CAD, Snail, and Slug) markers in primary cultures of adult human liver cells (*n* = 4, P2–4) compared to intact adult human liver tissue (*n* = 4). The results are normalized to *β-actin* expression within the same cDNA sample and presented as the relative levels of the mean ± standard deviation of primary cultures versus liver tissue. *P < 0.01.

**Figure 2:**
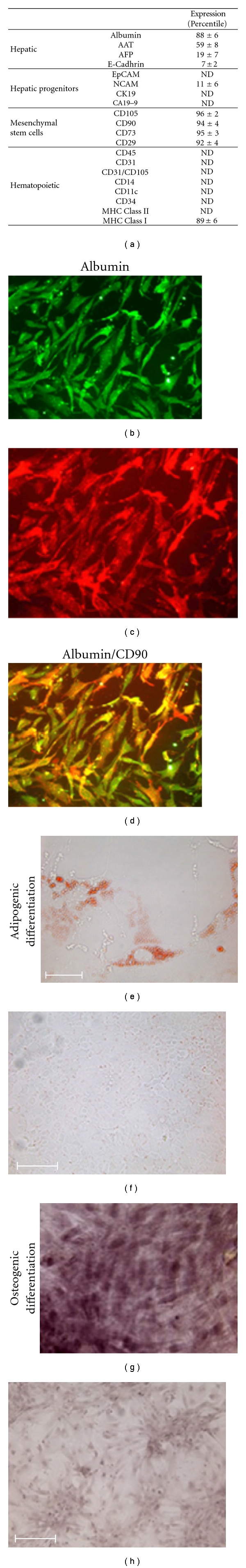
Primary adult human liver cell cultures express hepatic and mesenchymal stem-cell markers and differentiate along adipogenic and osteogenic lineages. (a) Adult human liver cells (P1–P7, *n* ≥ 5) were characterized for the expression of specific surface and intracellular markers of hepatic, hepatic progenitor, mesenchymal, and hematopoietic lineages by flow cytometry. (b–d) Double immunofluorescent staining of human liver cells (P7) for albumin and CD90. Nuclei were stained by DAPI (blue). Original magnification ×20. (e) Liver cells at P4 were induced to differentiate toward the adipogenic and (g) osteogenic lineages using the specific differentiation cocktails for 21 days in culture. Control cells in regular cell culture media are shown in (f) and (h). Samples were fixed and stained with Oil Red (adipogenic lipids, (e), (f) or Alizarin (osteogenic calcium, (g), (h)). Original magnification ×10.

Adult human liver cells can be propagated *in vitro* for roughly 20 passages; after an initial 2-week lag, the cells proliferate at a constant rate (Figure 1(a)) [14–17]. In addition to proliferation, a gradual and partial decrease in mature hepatic characteristics is observed [30]. A comparison of the gene expression profiles of primary cultures of adult human liver cells (passages 2–4), and the original intact tissues revealed changes in the repertoire of expressed genes (Figures 1(b) and 1(c)). Human liver-derived cells in culture undergo dedifferentiation, manifested as decreased expression of numerous adult hepatic markers and increased expression of immature and endodermal markers (Figure 1(b)) [30]. Previously, it was reported that hepatic dedifferentiation and downregulation of mature hepatic markers occurs rapidly, within 24 hours in culture [35]. Our data support that as the reduction was detected at any time point analyzed (P0–25 in culture). Despite the massive downregulation of adult hepatic markers, 88 ± 6% of the liver cells in culture maintains albumin expression and production, as demonstrated by flow cytometry and immunofluorescence (Figures 2(a) and 2(b)). Approximately 60% of the cells in culture were positive for another adult hepatic marker *α*-anti-trypsin (AAT) inhibitor (Figure 2(a)) though at lower levels than in the intact organ [30]. Twenty percent of liver cells in culture express the hepatic fetal marker alpha-fetoprotein (AFP), but none express the hepatic progenitor marker EpCAM or duct cell markers CA19–9 or CK19 [28, 36, 37]. Taken together, these results suggest that the proliferating cells in culture may not represent populations of hepatic stem cells. 

Liver cells in culture expressed lower levels of the epithelial marker E-CAD and higher levels of N-CAD compared to intact liver tissues (Figures 1(c) and 2(a)). Such a switch in cadherin expression usually characterizes an epithelial to mesenchymal transition (EMT) process, which also occurs to pancreatic islet cells in culture [38]. In addition to the switch in cadherin expression levels, expression of the transcription factors Snail and Slug was activated, which further strengthens the notion that proliferating human liver cells in culture may undergo an EMT process (Figure 1(c)). 

To further uncover the nature and properties of liver-derived cells in *in vitro* culture, we analyzed the expression of markers that characterize mesenchymal cells. Cellular characterization of the cells in increasing passages indicated that most liver-derived cells in culture express several mesenchymal stem cell (MSC) markers, including CD105, CD90, CD73, and CD29 (Figures 2(a) and 2(c)). Double immunostaining demonstrated the colocalization of hepatic and MSC markers within the same cells (Figure 2(d)). Hematopoietic marker expression was not detected in the human liver-derived cultures (Figure 2(a)). These data suggest that liver-derived cells in culture coexpress hepatic and general MSC markers but may not represent a known hepatic progenitor population, as neither CK-19 nor EpCAM expression is detected [28, 36, 37].

### 3.2. Developmental Plasticity of Liver-Derived Cells in Culture

The MSC markers present on most liver-derived cells in culture motivated us to analyze whether the cells also exhibit cellular plasticity. Human liver cells at passage 4 were cultured for 21 days under defined differentiation conditions known to activate osteocytes and adipocytes among BM-derived MSCs (Figures 2(e), 2(f), 2(g), and 2(h)). Indeed, human liver cells cultured in adipogenic differentiation medium exhibited lipid-containing droplets visualized by Oil Red staining (Figure 2(e)). Cells cultured in osteogenic differentiation media developed calcium deposits visualized by Alizarin Red staining, which is characteristic of osteogenic differentiation (Figure 2(g)). These data indicate that the liver-derived cells that propagate *in vitro* acquire developmental plasticity, which may allow them to differentiate along alternate developmental fates in response to applied growth and differentiation conditions. The developmental plasticity these cells exhibited may provide a partial explanation of their capacity to acquire a *β*-cell phenotype in response to ectopic pancreatic transcription factor expression.

### 3.3. Liver-Derived Cells Cultured *In Vitro* Do Not Induce Tumors in Immune Deficient Mice *In Vivo*

 The epithelial-mesenchymal transition in liver has been suggested to be related to invasiveness and metastatic potential in mouse and human cancers [39, 40]. Moreover, BM-derived mesenchymal stem cells have been demonstrated to exhibit tumorigenic capacity upon *in vivo* implantation [41–43]. Therefore, we analyzed whether the implantation of adult human liver-derived cells potentially generates tumors *in vivo* upon transplantation in immune-deficient mice. Adult human liver-derived cells at passage 4 were injected subcutaneously into both flanks of nude mice (1 × 10^6^ cells per injection, 5 mice per group, 2 transplantations per mouse) and tumor growth monitored weekly. Transplantation of tumor-derived cells, such as MDA-MB 231 or Panc-1 cells, resulted in large tumor formation 3–6 weeks after implantation [34], but none of the mice implanted with liver-derived cells developed visible tumors over 4 months. After 17 weeks, the mice were sacrificed and the area of injection examined. No traces of the cells were identified in the specimens. These data suggest that despite the cellular plasticity manifested earlier, dedifferentiated human liver cells have mesenchymal characteristics but may not carry a risk of uncontrolled cell proliferation or tumor formation. 

### 3.4. Irreversible Tracing of Albumin Expression

**Figure 3:**
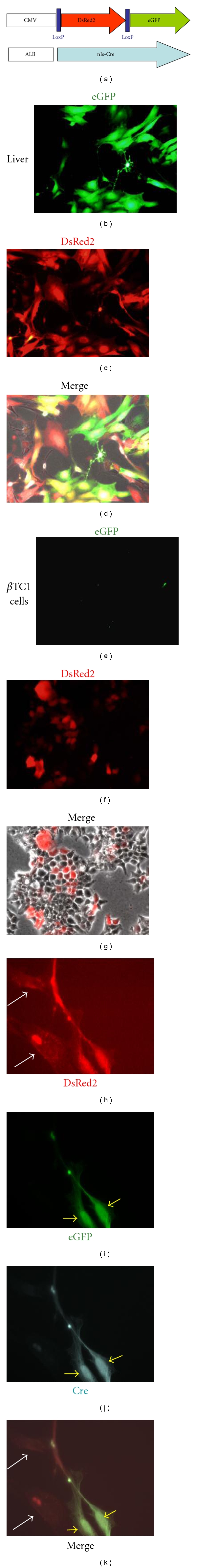
Irreversible labeling for albumin expression using the dual lentivirus system (lineage tracing). (a) Schematic presentation of the lentivirus vectors. (b–d) Adult human liver cells or (e–g) *β*-TC1 cells were infected with the reporter virus in combination with ALB-Cre virus. Liver cells were imaged 10 days after infection for DsRed2 (red) or eGFP (green) autofluorescence (original magnification ×10). (h–k) Immunostaining of adult human cells infected with both viruses for Cre (blue, (j)), eGFP (green, (i)), and DsRed2 (red, (h)). (k) Merged image of all stainings. White arrows indicate DsRed2-positive cells. Yellow arrows indicate eGFP and Cre double positive cells. Original magnification ×60.

The adult human-derived liver cells characterized above were reported in the past to undergo cellular reprogramming and generate insulin-producing cells upon ectopic expression of the pancreatic transcription factor PDX-1 [14, 16, 17, 30]. However, because only a fraction of PDX-1 expressing cells become insulin positive [14, 16], we sought to analyze whether the insulin-producing cells are generated from cells that originally express albumin or a yet unidentified side population of stem-like cells that may be enriched in the hepatic-derived primary cultures. Because PDX-1 turns off the hepatic repertoire of gene expression [30], we irreversibly tagged albumin expression prior to PDX-1 treatment. Human liver cells at passages 1–3 were coinfected by a dual lentivirus system modified from Russ et al. [33]. This lentivirus system included the CMV-loxP-DsRed2-loxP-eGFP (R/G) reporter [33] and an additional lentiviral vector carrying the expression of Cre recombinase under the control of the albumin promoter (ALB-Cre, Figure 3(a)). R/G treatment resulted in DsRed2, but not eGFP, expression in 84.1 ± 3.1% of cells. The albumin promoter activates the expression of Cre recombinase only in albumin-positive cells. Cre recombinase cleaves the “floxed” DsRed2, allowing the constitutive expression of eGFP under the same CMV promoter (Figure 3). The dual lentivirus system exhibited a high level of specificity; no eGFP-positive cells were detected in non-liver cells, such as pancreatic *β*TC1 cells (Figures 3(e), 3(f), and 3(g)), and Cre recombinase expression colocalized with albumin (Figures 3(h), 3(i), 3(j), and 3(k)). Because most adult human liver cells express albumin (88 ± 6%, Figure 2(a)), we expected that the majority of cells infected by both lentiviruses would have activated Cre recombinase and become eGFP-positive. The efficiency of infection with a single lentivirus was *∼*85%, and double infection resulted in 72% eGFP-positive cells, using CMV-Cre as control. Adult human liver cells infected with the two-lentivirus system (R/G and ALB-Cre) resulted in 69.4 ± 7.6% eGFP-positive cells within ten days of infection, with few cells expressing both eGFP and DsRed2 protein (Figure 3(d)). Colabeling with eGFP and DsRed2 likely reflects the activity of the albumin promoter and the relatively long half-life of the DsRed2 protein (*t*_1/2_   = 4.5 days), such that the DsRed2 protein can be detected even 1-2 weeks after the DsRed2 gene is no longer expressed [33]. Only 9.3 ± 5.4% of the cells remained irreversibly positive for DsRed2. These cells, in part, represent an incapability to activate albumin expression (about 2%-3%) and/or cells infected only with the reporter vector CMV-loxP-DsRed2-loxP-eGFP but not by the ALB- Cre lentivirus vector.

### 3.5. Characterization of Cells Irreversibly Tagged for Albumin Expression by eGFP

**Figure 4:**
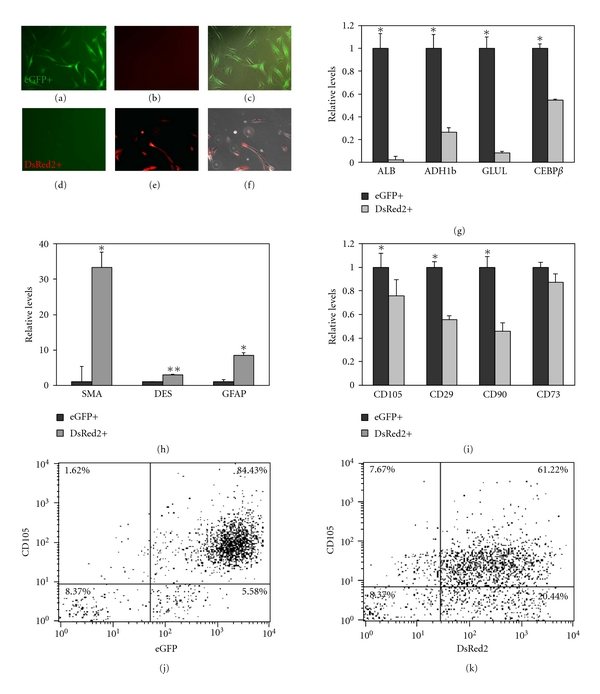
Isolated cultures of eGFP-positive cells express hepatic markers. Adult human liver cells were infected with the reporter virus and ALB-Cre virus. Two weeks later, eGFP-positive and DsRed2-positive cells were separated using a cell sorter. The eGFP-positive (a–c) and DsRed2-positive (d–f) cells were separately cultured and imaged 24 hours later for eGFP ((a) and (d), green) and DsRed2 ((b) and (e), red) fluorescence. (c and f) eGFP and DsRed2 merge images. Original magnification ×20. Quantitative RT-PCR in eGFP-positive cells compared to DsRed2-positive cells using mRNA extracted 2 weeks after sorting of (g) hepatic (albumin, ADH1b, GLUL, and CEBP*β*), (h) stellate cell (SMA, desmin, and GFAP), and (i) mesenchymal stem cell (CD105, CD29. CD90, and CD73) marker expression. The results were normalized to *β-actin* expression within the same cDNA sample and presented as the relative levels of the mean ± standard deviation of DsRed2-positive versus eGFP-positive cells, *n* = 6 from 3 different cultures. *P < 0.01, **P < 0.05. (j) The expression of the mesenchymal marker CD105 was analyzed in the eGFP and (k) DsRed2-positive cells by flow cytometry.

eGFP-labeled liver cells were separated from DsRed2-positive cells by FACS-Sorter and cultured separately for several passages (Figures 4(a), 4(b), 4(c), 4(e), and 4(f)). Two weeks after sorting (3 passages), less than 3% of DsRed2-positive cells were detected among the eGFP-positive population, the vast majority of which were colabeled by eGFP (data not shown), suggesting a highly purified culture of albumin-positive, eGFP-labeled cells. 

The eGFP and DsRed2 populations exhibited distinct hepatic marker expression. In addition to increased expression of the albumin gene in eGFP-positive cells (Figure 4(g)), additional adult hepatic markers, such as ADH1b, GLUL, and the transcription factor CEBP*β*, were expressed to higher levels compared to the DsRed2-positive cells (Figure 4(g)). However, the expression level of the hepatic genes in eGFP-positive cells was lower than that of cells at passage 2–4 (Figure 1(b)), further suggesting an ongoing dedifferentiation process which occurs in culture with time. The expression of immature or progenitor markers, such as AFP and CK19, was similarly detected in both groups (data not shown). In contrary, the DsRed2-positive cells were enriched for *α*SMA, desmin, and GFAP expression compared to the eGFP-positive cells (Figure 4(h)). *α*SMA, desmin, and GFAP are typically considered to be hepatic stellate cell markers [44]. Although stellate cells are considered mesenchymal cells [45], they do not usually express the mesenchymal marker CD90. The fact that DsRed2 cells also expressed *α*SMA, desmin, and GFAP but low levels of CD90 (Figure 4(i)) suggests that indeed, the DsRed2 population of cells may include the hepatic stellate cell population. Flow cytometry and immunofluorescence confirmed that each of the isolated eGFP-positive cells expressed albumin, CD105, and CD90 at higher levels than DsRed2-positive cells (Figures 4(j) and 4(k) and data not shown). 

### 3.6. Ectopic PDX-1 Expression Induces Cellular Reprogramming

**Figure 5:**
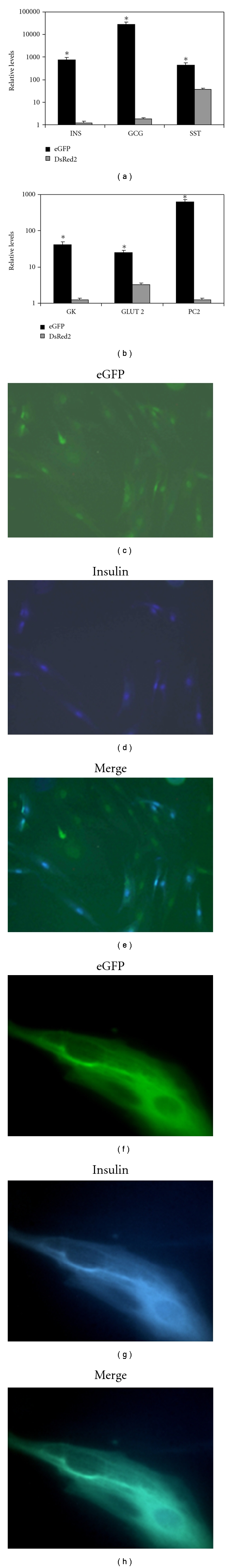
Ectopic PDX-1 expression induced pancreatic differentiation mainly in eGFP positive liver cells. eGFP-positive cells and DsRed2-positive cells were treated with *Ad*-PDX-1 and soluble factors for 5 days. Quantitative RT-PCR analysis of (a) pancreatic hormones (INS, GG, and SST), and (b) *β*-cell specific genes (GK, GLUT2, and PC2) in eGFP-positive cells and DsRed2-positive cells (*n* = 2 p7–9). The results are normalized to *β-actin* expression within the same cDNA sample and presented as the relative levels of the mean ± standard deviation of PDX-1 treated cells versus control Ad-*β*-gal treated cells. *P < 0.01. (c–h) Double immunofluorescence analysis of GFP with insulin in *Ad*-PDX-1 treated eGFP-positive liver cells. Original magnification ×20 (c–e) and ×60 (f–h). Five hundred cells were analyzed under the fluorescent microscope for eGFP, DsRed2, or insulin in three different cultures.

Next, we sought to analyze which of the two liver cell populations preferentially support PDX-1-induced reprogramming, manifested as induced insulin production. eGFP-positive cells and DsRed2-positive cells were separately treated with *Ad-PDX-1* and supplemented with soluble factors as previously described [16]. Ectopic PDX-1 expression resulted in a significant decrease in albumin gene expression (data not shown) [30]. However, ectopic PDX-1 expression did not affect the expression of CD105 and CD90 in the separate cultures (data not shown). Ectopic PDX-1 expression in eGFP positive cells activated the expression of pancreatic hormones gene expression (INS, GCG, and SST; Figure 5(a)), the expression of genes involved in *β*-cell glucose sensing (GLUT-2, GK), and prohormone processing (PC2) (Figure 5(b)). Only the expression of somatostatin was significantly activated in DsRed2 positive cells. Moreover, PDX-1 treatment resulted in insulin production, which mainly colocalized with eGFP; 17.6 ± 3.5% of eGFP-positive cells coexpressed insulin (Figures 5(c)–5(h)). In contrast, only 3.35 ± 2.1% of DsRed2-positive cells was also positive for insulin in response to a similar reprogramming protocol (data not shown). Because about 28% of the DsRed2-positive cells could have been positive for albumin but not eGFP, the actual percentage of insulin-positive cells generated in albumin-negative human liver cells is even lower than 3.35 ± 2.1%. Taken together, these data suggest that albumin-positive cells represent the vast majority of liver cells that undergo reprogramming along the pancreatic lineage.

## 4. Discussion

Using a genetic lineage-tracing approach, we demonstrate for the first time that the insulin-producing cells induced by ectopic expression of the pancreatic transcription factor PDX-1 in human liver cells mainly originate from albumin-positive cells. The proliferating adherent human liver cells express both adult liver and mesenchymal cell markers and possess a considerable level of developmental plasticity (Figures 1 and 2). 

Most liver-derived cells in the primary culture express mesenchymal characteristics (Figure 2). The origin of these hepatic MSC-like cells is not clear; they could represent liver cells that underwent EMT, or the cells may represent a preexisting population of stem-like cells with self-replication capacity, which normally serve as hepatic progenitor cells. The precise combination of markers that characterize human hepatic progenitor cells is controversial [29]; thus, it is complicated to completely rule out the possibility that our culture conditions promoted the amplification of pre-existing human hepatic stem-like cells. However, the comprehensive characterization of the primary culture of the human liver-derived cells seems to favor the option that insulin-producing cells are generated in dedifferentiated liver cells that underwent an EMT process, meaning that such cells, most likely, do not exist in the intact organ. This conclusion is based on several lines of evidence. First, 88% of the cells in culture are not only positive for albumin, but the albumin-positive cells also express higher levels of other adult hepatic markers, which is not expected to occur in pluripotent stem-like cells. Second, EpCAM is agreed upon as being a hepatic progenitor marker and is not expressed in our human-derived cells. Third, the only cells in the intact adult liver with mesenchymal characteristics are stellate cells. However, stellate cell marker expression was low in general and occurred mainly in the albumin-negative population, which had lower reprogramming efficiency (Figure 4(h)). Taken together, these data may suggest that the cells populating the majority of human liver-derived primary culture are not residents of the intact organ that underwent preferential proliferation. On the other hand, our human liver-derived primary culture exhibits several EMT characteristics, including specific mesenchymal marker expression (Figures 1 and 2), decreased E-CAD associated with increased N-CAD expression (Figure 1(b)), and the activation of Snail and Slug expression, the zinc finger transcription factors known to control the EMT process (Figure 1(c)) [40, 46]. 

Epithelial to mesenchymal transition is a common process that epithelial cells undergo upon *in vitro* culture [47–49]. *In vivo*, EMT plays a key role in morphogenic changes during embryonic development, wound healing/tissue regeneration, and neoplasia [25, 26, 30]. Several groups have demonstrated that the process is associated with the downregulation of epithelial gene expression and activation of mesenchymal gene expression [47–49]. EMT induction *in vitro* has been suggested in cultured thyroid cells [50] and adult human islets upon entrance into the cell cycle [51]. More recently, direct evidence of EMT in cultured adult primary *β*-cells was demonstrated using genetic lineage tracing [38]. The capacity of adult parenchymal liver cells to give rise to insulin-producing cells is further strengthened by *in vivo* studies. Direct administration of PDX-1 in mice *in vivo* suggests that the insulin-producing cells are primarily generated in parenchymal cells close to the central veins [8, 11, 25]. Hepatic pericentral cells are suggested adult, terminally differentiated, while adult hepatic stem cells mainly reside in periportal areas of the liver and in the Canals of Hering [22]. While these observations need to be directly analyzed by a lineage tracing approach, they suggest that the reprogramming process does not primarily take place in *bona fide* adult hepatic stem cells. 

Despite the uniform morphology of the human liver-derived cells *in vitro*, the cells seem to maintain different phenotypes with regard to albumin and the expression of other adult hepatic markers, which may correlate with distinct reprogramming capacity. Using irreversible lineage tracing for albumin promoter activity, we present supporting evidence that insulin-positive cells induced by ectopic PDX-1 expression are preferentially generated in cells that are originally albumin-positive (Figure 5). In a recent paper, Dan et al. [28] isolated human hepatic progenitor cells that carry the capacity to differentiate along multiple hepatic lineages, including mesenchymal cells. However, these cells did not express albumin. The colocalization of albumin expression with mesenchymal markers suggests that the reprogramming process of liver to pancreas does not occur in such cells but in hepatic dedifferentiated cells [30], which undergone an EMT process. 

Because we also detected a small number of insulin-positive cells among the DsRed2-positive cells, we cannot rule out the possibility that nonalbumin-positive cells present in our culture underwent reprogramming and contributed to the insulin-producing cell population but with lower reprogramming efficiency. 

The fact that the special mesenchymal cells coexpressing albumin do not induce tumors in immune-deficient rodents conveys a substantial safety advantage over using other progenitor cells in tissue engineering approaches [52, 53]. By contrast, other mesenchymal cells derived from bone marrow have been documented in numerous studies to carry a tumorigenic potential under similar conditions [41–43].

The data generated in this study provide a better understanding of the nature of primary human liver cell culture, which is capable of undergoing pancreatic transcription factor-induced reprogramming. Further dissection of subpopulations of cells within the liver-derived, albumin-positive mesenchymal cells will allow us to identify the specific characteristics of liver cells, which are predisposed to reprogramming to allow a substantial increase in the efficiency of the reprogramming process along the pancreatic lineage. 

## 5. Conclusion and Summary

The present study suggests that the insulin-producing cells induced by ectopic expression of the pancreatic transcription factor PDX-1 in human liver cells mainly originate from albumin-positive cells. The proliferating adult human liver cells in culture acquire mesenchymal characteristics and a high level of cellular plasticity. However, while most liver cells in culture possess mesenchymal-like characteristics, insulin production is induced in albumin positive cells, which are enriched for adult hepatic markers expression. Identification and the characterization of cells prone to reprogramming along the *β*-cell lineage is expected to increase the reprogramming efficiency. It may allow developing controlled and reproducible reprogramming process, by overcoming the pronounced heterogeneity of cells in the primary cultures.

Reprogramming liver to pancreas offers the access to an abundant source of “self-tissue”. The approach obviates the shortage in tissue availability from cadaveric donors and the need for antirejection treatment, allowing the diabetic patient to be the donor of his own therapeutic tissue.

## Supplementary Material

The primer pairs used in this study are listed in supplemental material data 2, annealing temperate of all primers is 60°c.
