# Supplementary material for: Human Liver Cells Expressing Albumin and Mesenchymal Characteristics Give Rise to Insulin-Producing Cells
Source: J Transplant. 2011 Aug 24;2011:252387. doi: 10.1155/2011/252387 (PMC3163017; doi:10.1155/2011/252387)
Supplement: Supplementary file 1 — The primer pairs used in this study are listed in supplemental material data 2, annealing temperate of all primers is 60°c. [file 252387.f1.pdf]

Supplement M1:

The antibodies used in this study are conjugated monoclonal antibodies against CD105 (1:100, *Fitzgerald Industries International*, Concord, MA), and CD90 (1:100, *BD PharMingen*, SanDiego, CA) and unconjugated monoclonal antibodies; rabbit anti human albumin (1:200 *Dako, Glostrup, Denmark*), guinea-pig anti human insulin (1:100 *Dako, Glostrup, Denmark*), rabbit anti GFP (1:100 *MBL, Woburn, MA*) and mouse anti GFP (1:100 *Santa Cruz Biotechnology*). Secondary antibodies were, anti-guinea pig IgG indodicarbocyanine (cy5), anti-guinea pig IgG aminomethylcoumarin (AMCA), anti-rabbit IgG Cyanine (cy2), anti-rabbit IgG indocarbocyanine (cy3), anti-mouse IgG Cyanine (cy2) conjugated antibodies (all 1:250, from *Jackson ImmunoResearch*, PA)

Supplement M2
Primers list,
Annealing temperate of all primers 60^0c

		F Primer	R Primer
ADH1b	NM_000668	GTCGGCTTGACACCATGATG	ACCCCTACGATGACGCTTGT
AFP	NM_001134	GTCCCTCCTGCATTCTCTGATG	TGGCCTTGGCAGCATTTC
ALB	NM_000477	AGGGTAAAGAGTCGTTATGCT	CAATCTCAACCCACTGTCAGCTA
CD90	NM_006288	TCCATCAGGTCCCCAATCC	CCCACCCCTCATCAGGAGTT
CEBPβ	NM_005194	GGAGCCCGTCGGTAATTTTAA	TCTGCATGTGCGGTTGGTT
CK18	NM_199187	CAGAGATCGAGGCTCTCAAGGA	GGCATCTACCTCCACGGTCAA
CK19	NM_002276	TGCGGGACAAGATTCTTGGT	GCAGGACAATCCTGGAGTTCTC
DES	NM_001927	GCGACAACCTGCTCGACGACCT	GCTCGGAAGGCAGCCAAATTGT
E-CAD	NM_004360	TGCCCCCAATACCCCAGCGT	ACGGTGGCTGTGGAGGTGGT
GCG	NM_002054	CCAAGATTTTGTGCAGTGGT	GGTAAAGGTCCCTTCAGCAT
GCK	NM_000162	CATCTCTGAGTGCATCTCCGACT	TCGCAGTGATGGTCTTCGTAGTA
GFAP	NM_002055	CGAGAAACCAGCCTGGACACCAA	TCCATCCCGCATCTCCACGGT
GLUL	NM_002065	GTTTGGCATGGAGCAGGAGTA	CCCATGCTGATTCCTTCACAA
GLUT2	NM_000340	TCCAGCTACCGACAGCCTATT	CCAGCCGTCTGAAA AATGCT
INS	NM_000207	GCAGCCTTTGTGAACCAACA	CGGGTCTTGGGTGTGTAGAAGAAG
N-CAD	NM_001792	CGATCTGCAGCCCCACACCC	TGGCCCCCAGTCGTTCAGGT
PC2	NM_002594	GAGAAGACGCAGCCTACACC	CTGCAAAGCCATCTTTACCC
SMA	MN_001613	GGAGAGCAGGCCAAGGGGCTATAT	AGGATTCCTGACAGTGCTTGGCTG
SST	NM_001048	ATGATGCCCTGGAACCTGAAG	GCCGGGTTTGAGTTAGCAGAT
β-actin	NM_001101	TTGCGACAGGATGCAGAA	CTCAGGAGGAGCAATGATCT
